# Removal and fate of pesticides in a farm constructed wetland for agricultural drainage water treatment under Mediterranean conditions (Italy)

**DOI:** 10.1007/s11356-021-16033-4

**Published:** 2021-09-02

**Authors:** Ilaria Braschi, Sonia Blasioli, Stevo Lavrnić, Enrico Buscaroli, Katia Di Prodi, Domenico Solimando, Attilio Toscano

**Affiliations:** 1 Department of Agricultural and Food Sciences, Alma Mater Studiorum University of Bologna, viale G. Fanin 44, 40127 Bologna, Italy; 2 GRIFA Gruppo di Ricerca Fitofarmaci e Ambiente, via Ospedale 72, 09124 Cagliari, Italy; 3 Central Laboratory of Conserve Italia Group, Conserve Italia Soc. Coop. Agricola, via P. Poggi 11, 40068, San Lazzaro di Savena, BO Italy; 4 Consorzio di Bonifica Canale Emiliano Romagnolo, via E. Masi 8, 40137 Bologna, Italy

**Keywords:** Imidacloprid, Dimethomorph, Glyphosate + AMPA, Dissipation kinetics, Soluble glyphosate-Ca complex

## Abstract

A non-waterproofed surface flow constructed wetland (SFCW), treating agricultural drainage water in Northern Italy, was investigated to gain information on the potential ability for effective pesticide abatement. A mixture of insecticide imidacloprid, fungicide dimethomorph, and herbicide glyphosate was applied, by simulating a single rain event, into 470-m-long water course of the SFCW meanders. The pesticides were monitored in the wetland water and soil for about 2 months after treatment. Even though the distribution of pesticides in the wetland was not uniform, for each of them, a mean dissipation of 50% of the applied amount was already observed at ≤7 days. The dissipation trend in the water phase of the wetland fitted (*r*^2^ ≥ 0.8166) the first-order model with calculated DT_50_ of 20.6, 12.0, 5.8, and 36.7 days for imidacloprid, dimethomorph, glyphosate, and the glyphosate metabolite AMPA, respectively. The pesticide behavior was interpreted based on the chemical and physical characteristics of both the substances and the water-soil system. Despite the fast abatement of glyphosate, traces were detected in the water until the end of the trial. The formation of soluble 1:1 complex between glyphosate and calcium, the most representative cation in the wetland water, was highlighted by infrared analyses. Such a soluble complex was supposed to keep traces of the herbicide in solution.

**Supplementary Information:**

The online version contains supplementary material available at 10.1007/s11356-021-16033-4.

## Introduction

The excessive use, the toxicity, and the environmental persistence of pesticides negatively affect the biodiversity of aquatic ecosystems and public health (Liao et al. 2020). Pesticides are one of the major threats to surface waters, including wetland environments and their communities. Any piece of information about persistence, soil-water-plant distribution, and transformation pathways of these substances in aquatic ecosystems is essential for future effective pesticide management.

Agricultural drainage water is one of the most important non-point sources of water pollution mainly due to the wide use of fertilizers and pesticides for crop productions. It is fundamental to intercept, to retain, and to treat drainage water before it is discharged into surface water bodies to limit the environmental spread of contaminants it contains.

Constructed wetlands (CWs) are an engineered technology mainly used for water and wastewater treatment that are designed for mimicking different processes occurring in natural wetlands (Gorito et al. 2017; Lavrnić et al. 2019; Nan et al. 2020). Two basic CW types are subsurface flow CWs, where water flows below ground, and surface flow CWs (SFCWs), where water flows across the plant roots and is in direct contact with the atmosphere.

CWs have been used for the treatment of various types of water such as domestic or industrial wastewater, road runoff, landfill leachate, agri-industrial and livestock effluents, liquid digestates, saline effluents, and agricultural drainage water (Lavrnić et al. 2020b; Lavrnić et al. 2019; Maucieri et al. 2016; Nan et al. 2020; Zhu and Bañuelos, 2017). With respect to other treatment technologies, CWs have lower environmental footprint, lower cost, and energy operation and are at easier integration with the environment (Fuchs et al. 2011; Ingrao et al. 2020; Si et al. 2020).

The ability of CWs to remove pesticides has been extensively documented (Butkovskyi et al. 2021; Fernández-Pascual et al. 2020; Liu et al. 2019; Matamoros et al. 2020; McMaine et al. 2020). To this end, the hydraulic residence time is an important factor for the process (Tournebize et al. 2017). Tang et al. (2016) showed that integrated recirculating CW was able to remove chlorpyrifos, endosulfan, fenvalerate, and diuron, and underlined the effect of plants in the dissipation process. In CWs, plant-microorganisms consortia and biofilms are essential for pesticide dissipation (Lv et al. 2017).

SFCWs are valuable systems to return water of ameliorated quality (Maillard and Imfeld, 2014; Mendes et al., 2018). In these systems, the contribution of aquatic plants to overall nutrient and pesticide dissipation through rhyzodegradation and uptake is a key factor (Boog et al. 2019; Cancelli et al. 2019; Sonkamble et al. 2019). The high surface area and reactivity of solid mineral and/or organic fine particles of wetlands are active components in retaining and transforming pesticides. On the other hand, they can also transport adsorbed compounds at long distances along waterways owing to possible outflow (Bento et al. 2018; Yang et al. 2015).

Only a few studies addressed the potential of full-scale CWs to retain (i.e., buffer/barrier effect) and to reduce (i.e., treatment effect) relevant concentrations of pesticides as it can be observed during extreme rain events or excessive irrigations (Pappalardo et al. 2016; Tsui and Chu, 2008).

In wetlands, the uptake and biotic transformation of pesticides by plants and microorganisms, as well as catalytic, hydrolytic, and photolytic transformations, are components of the cumulative dissipation process performed by these aquatic systems as a whole. At a full scale, the quantification of each of these simultaneous and combined dissipation mechanisms is extremely difficult to gain. From an environmental point of view, a clear indication of the advancement of the pesticide dissipation process inside the wetland can be obtained by measuring the pesticide content in the soil and water compartments.

Neonicotinoid insecticide imidacloprid, morpholine fungicide dimethomorph, and phosphonoglycine herbicide glyphosate are pesticides commonly used at agricultural farms to protect and to increase the yield of several crops. Imidacloprid is highly toxic to birds and honeybees, dimethomorph shows high toxicity for mammals (rats), and glyphosate has moderate toxicity for mammals (rats) and fish (Lewis et al. 2016). The high persistence of the neonicotinoid insecticides in water outflowing SFCWs can be a threat to waterways and insects as highlighted by EU Regulation No 485/2013 (ec.europa.eu, 2020). These pesticides are frequently detected in Italian surface and groundwater (Report ISPRA 2018).

This study evaluated the potential of an Italian full-scale SFCW, which is currently used for the treatment of agricultural drainage water, to act as an ecological practice for the retention and removal of a mixture of imidacloprid, dimethomorph, and glyphosate at high concentrations (ca. 1 mg L^−1^ each) as a critical case that could be observed under unfavorable conditions (e.g., extreme runoff from surrounding agricultural fields and/or unsuitable agricultural practices as incorrect accidental/voluntary dumping of tank washings or commercial formulations). The pesticides were introduced simultaneously, as a mixture of commercial formulations, into the wetland through a single input. The efficacy of the system in the abatement of the pesticides was derived by a 2-month observation of their distribution between soil and water compartments. For each pesticide, the environmental behavior in the wetland was then interpreted based on the chemical and physical characteristics of the substance and of the soil-water system.

## Materials and methods

### Description of the SFCW

**Figure 1.**
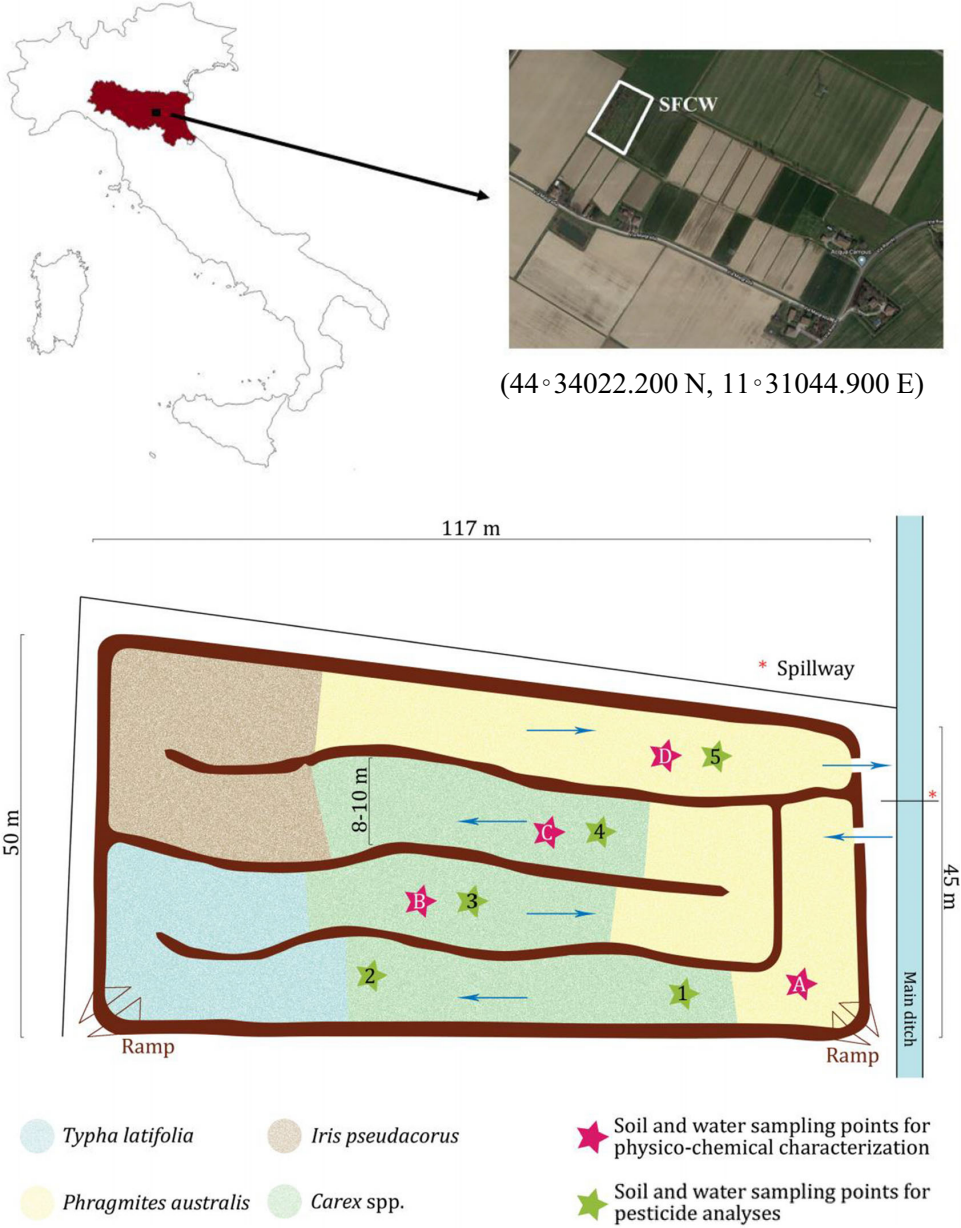
Map, GPS coordinates, and scheme of the surface flow constructed wetland (SFCW) located in Northern Italy. Blue arrows indicate the water flow path inside the system

The study was conducted in Northern Italy at the SFCW of the Acqua Campus of Canale Emiliano Romagnolo Land Reclamation Consortium (CER), located in Budrio municipality (Emilia-Romagna region). Since its construction in 2000 at the 12.5-ha experimental agricultural farm of CER, the SFCW was treating drainage water coming from the farm area (Lavrnić et al., 2018) where fruit trees, vegetables, and cereals are grown. The system has a surface area of about 3700 m^2^ and is divided into four meanders (Figure 1) that create a 470-m-long water course with a maximum water volume capacity of about 1500 m^3^. The inflow into the SFCW depends on the presence of rain, on soil retention capacity, and on related runoff in the drained area.

Despite the SFCW has been artificially built, currently, after 20 years of operation, it is perfectly integrated into the farm area and can be considered as a semi-natural system that regulates deposition, erosion, and (re-)distribution of sediment, flow paths, preferential and stagnant zones, and its own flora and fauna. This integration could be better seen through the high inhomogeneity of plant species within the wetland, as sketched in Figure 1. The system was fully but irregularly inhabited mostly by common reed (*Phragmites australis*), cattail (*Typha latifolia*), sedge (*Carex* spp.), and yellow iris (*Iris pseudacorus*). In the light of these non-homogeneous characteristics, in the terms of hydraulic behavior and removal efficiency, the SFCW might be better considered as a whole (i.e., like a “black box”) than a plug-flow or even a completely mixed reactor.

The SFCW has irregular flow depending on the presence of rain and drainage water. Like other wetland systems used in agricultural contexts, it is not waterproofed and has an overall soil infiltration rate of about 0.3 mm h^−1^ (Lavrnić et al., 2020a). The infiltration can be considered almost negligible for the purposes of the present study in that USDA recommends waterproofing of soils with an infiltration rate ≥15 mm h^−1^ (USDA, 2007).

The meteoric water from the entire farm area is drained into a ditch from where it is pumped into the wetland once the water level reaches a certain threshold. The outflow from the system is regulated by gravity and occurs after the level of water inside the SFCW reaches 40 cm.

The SFCW is equipped with two mechanical flow meters for the measurement of inlet and outlet water volumes and an automatic sensor for the measurement of water level inside the system. All the collected hydraulic data are managed and recorded by a centralized control system. Rainfall is measured by a tipping-bucket rain gauge located 500 m far from the wetland.

### Chemical and physical characterization of the wetland soil and water

On October 11, 2017, 1 week before the pesticide distribution, soil cores 60 cm long were sampled from four positions (positions A–D as indicated in Figure 1). Each sample core was divided into 5 portions at 0–5, 5–15, 15–30, 30–45, and 45–60 cm of depth.

Soil samples were characterized for pH, electrical conductivity (EC), moisture, texture, CaCO_3_ content, total organic carbon (TOC), total nitrogen (TN), and other macro- (P, K, Ca, Mg, and S) and micro-nutrients (Fe, Zn, Cu, Mg, B, Mo, and Ni). Conditions and methods for soil characterization are reported in Lavrnić et al. (2020b).

Samples (1 L each) of the SFCW water are collected at positions A–D (Figure 1) on October 18, 2017 (namely, 24 h after the pesticide introduction and at the end of the pesticide distribution along the wetland meanders by water inflow, see the “Experimental design and conditions” Section). The water samples were mixed, and the gathered sample was analyzed for pH, EC, TOC, TN, and micro- and macronutrients. Conditions and methods of water characterization are reported in Lavrnić et al. (2020b).

### Pesticides

Three pesticides were selected: insecticide imidacloprid [*N*-{1-[(6-chloro-3-pyridyl)methyl]-4,5-dihydroimidazol-2-yl}nitramide], fungicide dimethomorph [3-(4-chlorophenyl)-3-(3,4-dimethoxyphenyl)-1-morpholin-4-ylprop-2-en-1-one] and herbicide ghyphosate [*N*-(phosphonomethyl)glycine]. Table 1 reports solubility, volatility, *n*-octanol/water partitioning coefficient (Kow), acid dissociation constant (pKa), and environmental persistence of the pesticides and AMPA [(aminomethyl)phosphonic acid], the main metabolite of glyphosate.

**Table 1:**
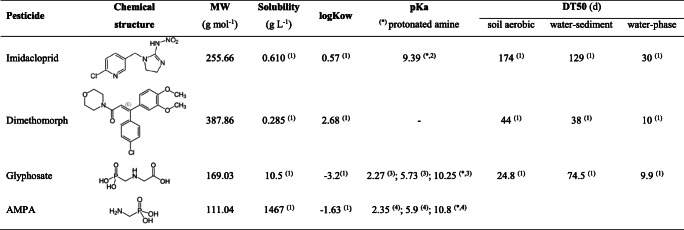
Chemical and physical properties and environmental persistence of the pesticides under investigation. ^(1)^ From Pesticide Properties Database (Lewis et al. 2016); ^(2)^ From ChemAxon https://chemaxon.com/; ^(3)^ Piccolo and Celano, 1994; ^(4)^ From ChemicalBook https://www.chemicalbook.com/productindex_en.aspx

The following commercial formulates of the pesticides were added to the CW: Corsario (soluble concentrate, 16.5–18.0% imidacloprid w/w, Scam), Forum R (wettable powder, 6% dimethomorph w/w, Basf), and Roundup Platinum (soluble concentrate, 44% glyphosate w/w, Monsanto).

### Experimental design and conditions

The pesticide mixture was added to the SFCW on October 17, 2017. Before the pesticide distribution, the level of water inside the SFCW was approximately 10 cm (corresponding to 369 m^3^).

According to the dilution indications given in the pesticide labels, Corsario (3.5 kg, corresponding to 621 g of imidacloprid), Forum R (10 kg, corresponding to 600 g of dimethomorph), and Roundup Platinum (2.7 kg, corresponding to 1188 g of glyphosate) were placed into a 0.5-m^3^ plastic container previously filled with 0.45 m^3^ of water and thoroughly mixed by a pump before its introduction into the CW at the inlet (Figure 1). In the container, the dissolution of imidacloprid and glyphosate was guaranteed by their soluble concentrate formulations, while the homogeneity of Forum R suspension was guaranteed by its formulation as a wettable powder. The introduction of the pesticide suspension (0.45 m^3^) in the wetland was immediately followed by the admission in the system of an additional volume of 428 m^3^ of water to ensure a proper initial distribution of pesticides inside the system. The water volume of 428 m^3^ was chosen to allow the pesticides suspension to reach approximately not more than two-thirds of the water course (position 4, Figure 1) to prevent any outflow and, thus, to protect the connected waterways.

The introduction of the pesticide suspension (0.45 m^3^), which lasted approximately 20 min, could be considered almost instantaneous if compared to the SFCW hydraulic residence time of 6.7 days (Lavrnić et al. 2020a), while the water inflow of the additional 428 m^3^ ended 20 h later. This volume is representative of an inflow that would occur in case of 20-mm rain event over the catchment area, as already assessed on the same wetland (Lavrnić et al. 2018, 2020b). Based on almost 20 years of recorded hydrological and meteorological data (not shown), these experimental conditions were designed to simulate, after an intensive inflow event, a period without precipitation and outflow and, thus, to represent the real operational conditions of the wetland.

**Figure 2:**
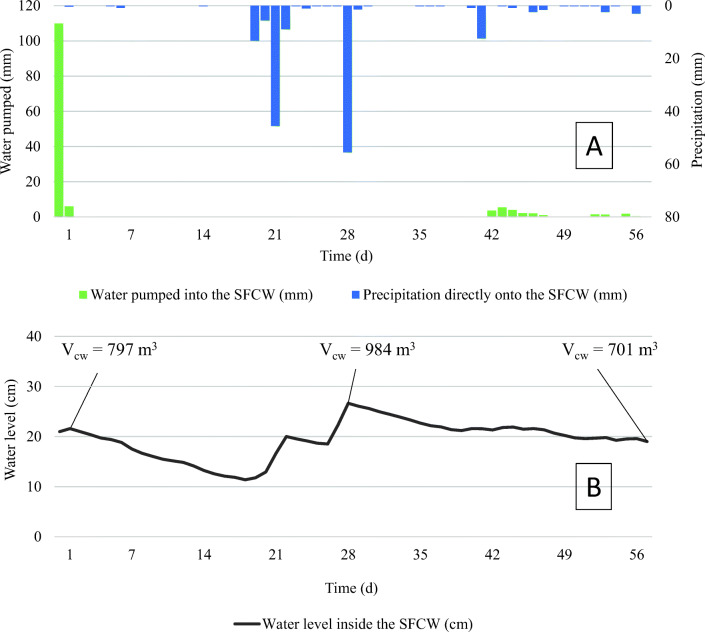
**A** Precipitation (mm) and pumped water (mm) that entered the SFCW during the 56 days of pesticide dissipation trial. **B** Water level (cm) of the SFCW during the same period. V_CW_, volume of the SFCW calculated multiplying the SFCW surface by the water level

After pesticide distribution, the SFCW water volume resulted about 797 m^3^ (369 + 428 + 0.45 m^3^), corresponding to a water level of 22 cm (Figure 2B). Here, the theoretical initial concentration of imidacloprid, dimethomorph, and glyphosate was 0.78, 0.75, and 1.40 mg L^−1^, respectively. Such high pesticide loads were introduced into the wetland to create a worst-case scenario and to assess the dissipation behavior of the wetland in such a critical situation.

These selected pesticide levels were about 50–60 times higher than dimethomorph and glyphosate peak maximum concentrations detected in Italian surface waters and about 1,100-fold higher for imidacloprid peak maximum concentrations (Report ISPRA 2018). It should be noted, however, that the pesticide concentrations in receiving surface waters are necessarily considerably lower than those present in farm drainage systems. In fact, pesticides are dissipated and diluted along the distribution water network—often several kilometers of pipelines and canals—before reaching the final recipient ecosystems, especially when the receiving water bodies are rivers with high flow rates.

The dissipation study lasted 8 weeks. In this period, the SFCW water level (Figure 2B) and the water inflow (Figure 2A) were closely monitored. After the pesticide distribution, the influent pumps were kept turned off for 6 weeks (until November 29, 2017). During this period, the only inflow was rainwater collected by the wetland surface. Such a long period without any inflow prevented further dilution of the pesticides and simulated routine operation of the system. Long periods without inflow are a normal operation mode in Mediterranean areas. Water drainage inflow into the system was allowed during weeks 7 and 8 (Figure 2A). The first outflow from the system occurred in February 2018, 4 months after the pesticide distribution, owing to major rain events.

The average daily temperature during the experimental period was recorded and is reported as a Supplementary Material in Figure SM.1.

### Soil and water sampling for pesticide analysis

Since the experimental conditions were designed so that pesticide suspension could not reach the outlet device, the sampling positions 1–4, where the presence of pesticides was expected, were placed approximately at the same distance along the path (Figure 1). The sampling position 5 was located near the outflow of the system to serve as a control point where pesticides were not expected to be found. The absence of pesticides at this position should protect the surrounding environment by their release from the wetland in the case of accidental events, as a heavy rain, that could cause the increase of water level in the system.

SFCW soil and water samples were collected on October 18 (i.e., at the end of the water inflow of 428 m^3^ into the wetland to distribute the pesticides along the meanders) and weekly for 8 weeks, to evaluate the pesticide concentrations. During collection and manipulation of soil and water samples, polypropylene (PP) plasticware was used because of the tendency of glyphosate and its main metabolite, AMPA, to interact with glass surfaces (Anastassiades et al. 2015). Measurable adsorption of imidacloprid and dimethomorph by the plastic bottles was previously excluded by laboratory tests.

Water samples were collected plunging 1-L plastic bottles in the wetland at positions 1–5 and were immediately frozen at −20°C until processing.

Single soil cores of 60 cm long were sampled from each position at day 1 and at weeks 1–4. Owing to the absence of detectable concentrations of pesticides in the soil profile below 5 cm, soil cores of 30 cm long were sampled in the following 5–8 weeks. Each core was divided into 0–5-, 5–15-, and 15–30-cm (and 30–45-, 45–60-cm, when sampled) portions. Each portion was placed in a plastic bag, stored at 5°C in a refrigerated container and immediately processed for analysis.

### Extraction and analysis of pesticides from soil and water samples

The collected soil samples were placed on a grid previously covered with filter paper and left to drain. Drained soil samples were homogenized by mechanical mixing. An aliquot (5 g) of each homogenized sample was placed into a Nalgene centrifuge tube (Thermo Fisher Scientific) and added with 5 mL of a milliQ H_2_O:CH_3_CN (HPLC grade, Sigma Aldrich) mixture in the ratio of 1:1 for imidacloprid and dimethomorph or with 5 mL CH_3_OH for glyphosate analysis, respectively. Then, each suspension was horizontally shaken for 10 min, sonicated for additional 10 min, and then centrifuged at 24000 *g* for 15 min. The supernatant was recovered and filtered at 0.45 μm with a nylon filter (Minisart Syringe Filter Sartorius). An aliquot of the solution was withdrawn and directly analyzed by HPLC.

Water samples were thawed at room temperature (RT) and sonicated for 5 min to dissolve precipitate eventually formed. An aliquot (100 mL) of each sample was filtered under vacuum at 0.45 μm with a nylon membrane filter (Sartorius) and directly analyzed by HPLC. Solvent extraction and subsequent concentration steps that are usually adopted for the quantification of traces or low levels of pesticides in water phase could be avoided due to the high concentrations of pesticides applied to the wetland.

An assessment of the recovery efficiency of the three pesticides from the SFCW soil-water system was performed at about 100 and 10% of the initially applied dose (namely, 1.0 and 0.1 mg L^−1^ for imidacloprid or dimethomorph and 1.5 and 0.15 mg L^−1^ for glyphosate). Detailed information on the pesticide recovery from the SFCW water and soil compartments are given as a Supplementary Material SM.1. The recovery efficiency of each pesticide from the SFCW soil-water system at both 100 and 10% of the initially applied dose was 96.5±0.6 and 95.1±1.0%, respectively, for imidacloprid; 98.0±7.0, and 96.9±8.8% for dimethomorph; 52.2±2.0 and 45.0±5.1% for glyphosate. The low values of glyphosate recovery efficiency were considered acceptable owing to its known fast binding in submerged soils (Bois et al. 2013).

The quantification of pesticides was performed at Central Laboratory of Conserve Italia Group by UHPLC ACCELA (Thermo Fischer Scientific) connected to a TSQ QUANTUM ACCESS mass spectrometer detector (Thermo Fischer Scientific). The setup of conditions adopted for the analysis is reported as Supplementary Material SM.2. The limit of quantitation (LOQ) and the limit of detection (LOD) for the three pesticides and AMPA in soil and water samples were 0.05 and 0.008 mg kg^−1^, respectively.

### Dissipation kinetics

The experimental mean concentrations of pesticides (average concentrations among 1–5 sampling positions) were used for modeling their dissipation in the SFCW water phase and in the soil-water system with time. The concentrations were initially fitted to the first-order exponential kinetics (ln[*C*]_0_/[*C*]_*t*_ *= kt*) and then to the bi-phase Hockey-stick model consisting of two sequential first-order curves identifiable by a breakpoint(ln[*C*]_0_/[*C*]_*t*_ = *k*_1_*t* for *t* ≤ breakpoint; ln[*C*]_0_/[*C*]_*t*_ =  *k*_2_*t* for *t* ≥ breakpoint) for the reason described in the following section.

For both models, according to the indications reported in the Generic guidance for Estimating Persistence and Degradation Kinetics from Environmental Fate Studies on Pesticides in EU Registration (EU DG-SANCO, 2006), the initial theoretical and the first sample field concentration of the pesticides could be included or not in the calculation based on the best fitting due to the occurrence of several phenomena far from the equilibrium (i.e., pesticide desorption from submerged plant biomass/biofilm) that likely take place at short contact times.

### Infrared analysis of water solutes-glyphosate complexes

Owing to the fast reaction of glyphosate with the wetland soil components and the occurrence of its traces in water for the entire duration of the experimental trial, the interactions of glyphosate with soil and water components were investigated by infrared spectroscopy.

Glyphosate (5 mg) was dissolved into 30 mL of wetland water previously filtered at 0.4 μm to give 1-mM final concentration. The water was immediately freeze-dried and the solid phase analyzed by IR spectroscopy. Similarly, wetland water (30 mL) was filtered and freeze-dried and the solid analyzed as a control.

Complexes of glyphosate with Ca, Na, or Cu ions at pH 8 were prepared as models for IR analysis. Glyphosate solutions (2 mM) containing Ca, Na, or Cu ions at the ratio of approximately 1:1 eq_(−)_:eq_(+)_ at pH 8 were prepared. Glyphosate (5 mg) was dissolved in 15 mL of milliQ water in the presence of the following: (i) Ca^2+^ (4.35 mg of CaCl_2_·2H_2_O); (ii) Na^+^ (1.75 mg of NaCl); (iii) Cu^2+^ (5.04 mg of CuCl_2_·2H_2_O). The pH of each solution was then adjusted to 8 with a few drops of 0.1 M NaOH. The solutions were immediately freeze-dried and the solids analyzed. IR analyses were performed on pellets of freeze-dried samples.

Samples of freeze-dried aqueous solutions (2.5 mg each) were thoroughly mixed with 15 mg of KBr (Sigma Aldrich, IR spectroscopy grade). Pellet of each sample was obtained by mechanical press (Specac) at ca. 7 tons cm^2^ and placed into an IR cell equipped with KBr windows permanently attached to a vacuum line (residual pressure 5×10^−2^ mbar). Finally, FT-IR spectra of the pellets were collected on a TENSOR 27 (Bruker) with 4-cm^−1^ resolution and 64 scans.

## Results and discussion

### Characterization of the SFCW water and soil

The full characterization of the SFCW water and soil is reported as a Supplementary Material (Tables SM.1 and SM.2, respectively). Briefly, the water had an alkaline pH of 7.96, a TOC of 15.7 mg L^−1^, and an EC of 0.426 mS cm^−1^. The main elements were as follows: Ca (56.4 mg L^−1^), Mg (15.1 mg L^−1^), Na (10.1 mg L^−1^), Cu (0.178 mg L^−1^), Fe (0.11 mg L^−1^), and Al (0.01 mg L^−1^). Sulfates and nitrates were 37.3 and 1.7 mg L^−1^, respectively. Before the pesticide addition, none of the pesticides was detected (LOD = 0.008 mg L^−1^) in the SFCW water.

In Table 2, only the characteristics of the 5-cm topsoil are shown owing to the absence of detectable value of pesticides at underlying soil depth as described in the following section. The mean textural analysis and the bulk density of the 5-cm topsoil indicated a silty clay loam compacted soil. The soil was alkaline (pH = 8.27 on average) and moderately calcareous with 14.4% of carbonates. The high variability of the topsoil structure parameters among the sampling positions (i.e., clay content in the range 27.5–41.3%) was explained because of the high inhomogeneity of plant species and density among the meanders and embankments and of the water level due to different sediment deposition rates. The variability of TOC (in the range 2.8–5.6%) and TN (0.24–0.43%) could be more related to the life cycle of the different aquatic plants, never harvested, inhabiting the systems. On the contrary, the decreasing trend of EC (from 607 to 487 μS cm^−1^) and carbonates (from 15.8 to 12.0%) in the SFCW soil samples from positions A to D could be due to the water volume collected and treated by the system. In fact, under normal functioning, and for the most part of the time, the first meanders contained more water than the last ones as the inflow is often not high enough to fill the whole system. Consequently, the soil enriched of salts at decreasing values from positions A to D. In addition, the decreasing trend of soil carbonates along the water course could be related to the activity of microbiota and aquatic plants (i.e., respiration and transpiration) that was augmented by the level of salts (and nutrients) in the water phase.

**Table 2:** Physical and chemical characteristics of the SFCW topsoil samples (0–5-cm depth) collected at A–D positions shown in Figure 1. Except for humidity, measure units are referred to soil dry weight. Relative analytical error <5% with the sole exception of pH (<1%)

**Parameter**	**Sampling position**				**Mean**	**STD**
	A	B	C	D		
Humidity [% on bulk]	21.4	22.5	23.5	19.4	21.7	1.8
Bulk density [kg m^−3^]	1900	2100	2100	2300	2100	163
pH (H_2_O)	8.30	8.19	8.42	8.17	8.27	0.12
pH (CaCl_2_)	7.75	7.81	8.05	7.59	7.80	0.19
Electric conductivity [μS cm^−1^]	606.85	593.80	510.75	486.70	549.52	59.71
Carbonates [%]	15.8	15.8	13.8	12.0	14.4	1.8
*Texture*						
Sand [%]	11.5	15.9	13.4	14.8	13.9	1.9
Silt [%]	61.0	42.9	53.6	57.7	53.8	7.9
Clay [%]	27.5	41.3	33.0	27.5	32.3	6.5
*Macronutrients*						
Total organic carbon [%]	3.6	4.6	2.8	5.6	4.2	1.2
Total nitrogen [%]	0.35	0.39	0.24	0.43	0.35	0.08
C/N ratio	10.4	11.9	11.7	13.0	11.8	1.1
Total phosphorous [mg kg^−1^]	710	600	610	730	663	67
*Other elements*						
Al [g kg^−1^]	34.4	33.1	35.7	32.8	34.0	1.3
Fe [g kg^−1^]	25.4	23.9	26.0	24.1	24.9	1.0
Cu [mg kg^−1^]	40.8	33.1	37.4	36.3	43.7	3.2

Before the pesticide application, none of the three pesticides was detected (LOD = 0.008 mg L^−1^) in the soil up to 60 cm of depth.

### Behavior of the pesticides in the SFCW soil-water system

The water volume of the wetland changed with time, as shown in Figure 2B, sensibly decreasing during the first 3 weeks (from 797 to 420 m^3^). The average water loss that occurred during the 8-week trial (around 0.24 mm h^−1^) was mainly consistent with the soil infiltration rate (Lavrnić et al., 2020a). The evapotranspiration rate could be considered negligible especially because the trial was conducted during the plant dormancy period. At week 4, the SFCW water volume increased to 984 m^3^ due to important precipitation, and then it decreased again to 790 m^3^ at week 6. Finally, in the last 2 weeks of experimentation (weeks 7–8), the pumps were turned on and 85 m^3^ of farm drainage water were admitted into the wetland.

The concentration of each pesticide in the first 5 cm of topsoil (mg kg^−1^ dw) and in the water (mg L^−1^) samples collected from each sampling position (positions 1–5, Figure 1) can be found in Figure SM.2 as a Supplementary Material. Although the SCFW was not waterproofed, the absence of pesticides at detectable levels at a soil depth below 5 cm highlighted the ability of the topsoil to retain the pesticides and, thus, to contain their downward movement during the water infiltration phase. Likely, the relevant content of total organic carbon (TOC 4.2% on average, Table 2) and the compactness of the silty clay loam topsoil impeded the pesticide migration along the soil profile. Although pesticide concentrations <LOD (0.008 mg kg^−1^ dw), eventually contained in soil portions below 5 cm, can be considered environmentally relevant, their contribution to the pesticide mass balance in the soil-water system could be considered negligible.

**Figure 3:**
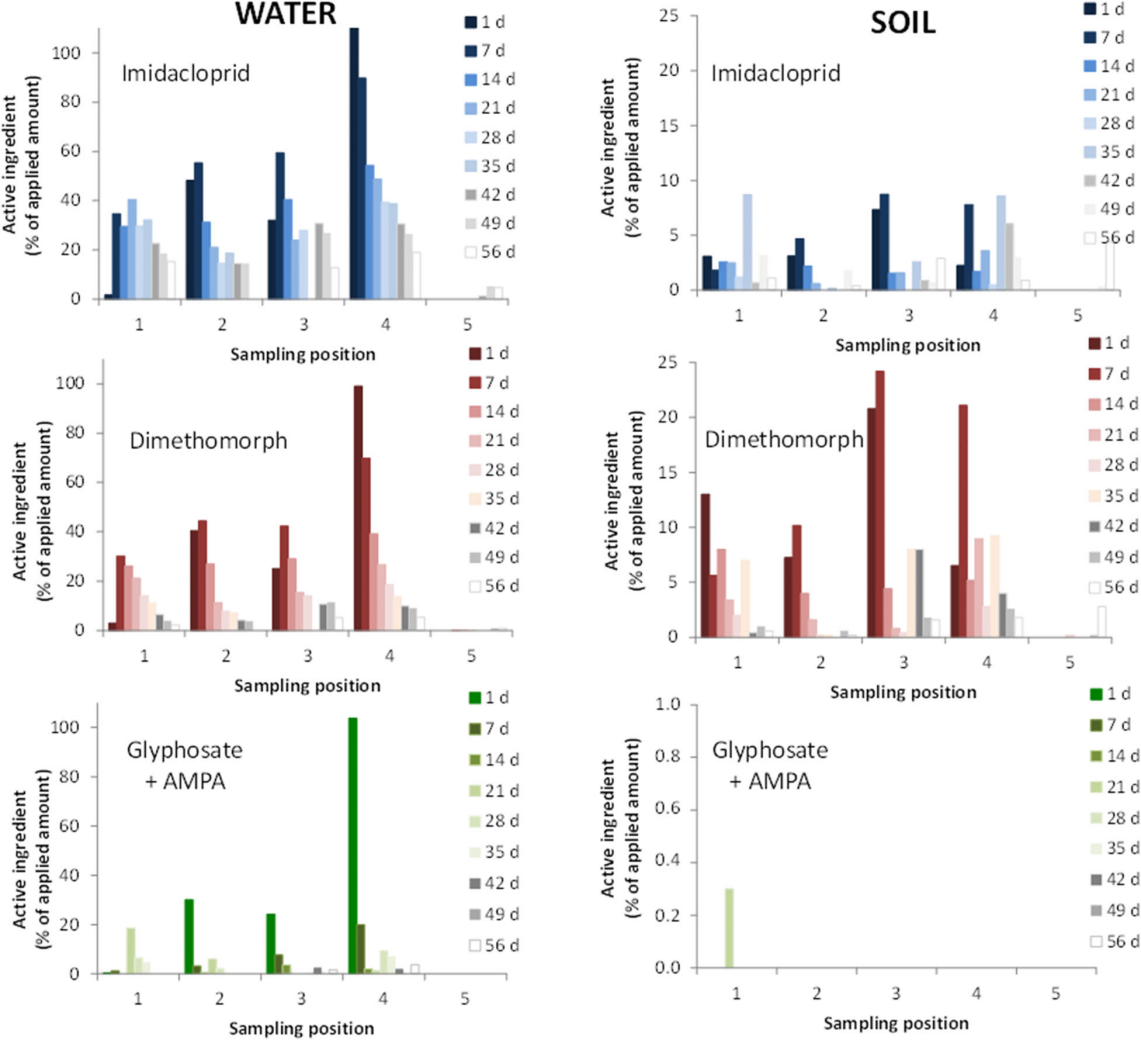
Imidacloprid, dimethomorph, and glyphosate+AMPA concentration in the SFCW water and soil, expressed as a percentage of the amount added into the SFCW, over 8 weeks of observation. Molar percentage of AMPA, the main metabolite of glyphosate, is calculated with respect to the moles of glyphosate applied to the wetland

**Figure 4:**
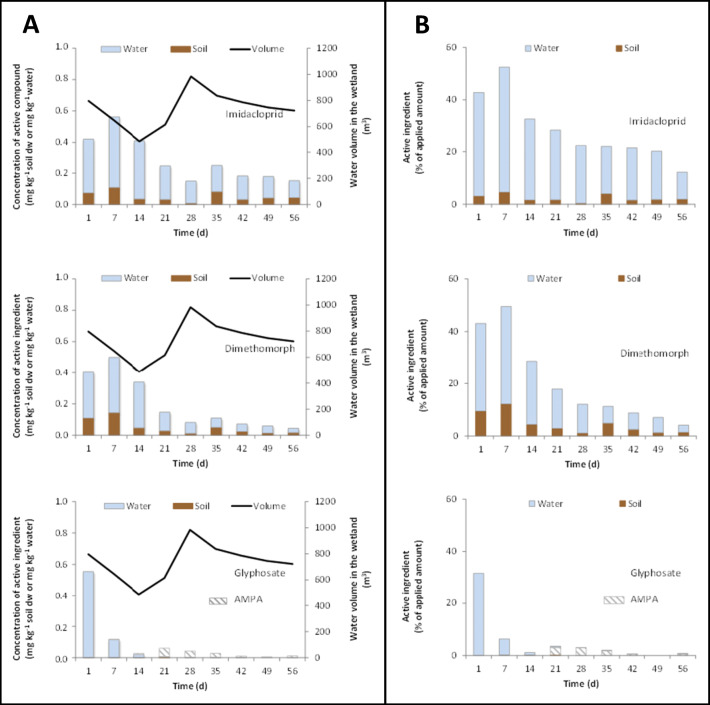
**A** Trend of the average concentrations among 1–5 sampling positions of pesticides and AMPA in the SFCW soil (mg kg^−1^) and water (mg L^−1^) with time. The total volume of SFCW water volume (m^3^) variable with time is also reported. **B** Trend of the average percentage among 1–5 sampling positions of initially applied pesticides and AMPA in the soil and water with time

In Figure 3, the pesticide concentrations, expressed as a mean percentage of the amount of each active ingredient initially added to the CW (100% at day 0), in the water and in the first 5 cm of topsoil, weekly sampled at positions 1–5, are reported. The expression of the percentage concentration enabled to better follow their dissipation in the varying water volume of the wetland, as well as their distribution between the soil and water compartments. In the figure, the molar concentrations of glyphosate and AMPA were summed up and the sum was expressed as a percentage of the glyphosate molar amount added into the wetland. This operation was done to align our data with the request of the current environmental regulation (e.g., ISPRA reports) to express glyphosate and AMPA as a sum. A separate balance for the two chemicals can be found in Figure 4, where the average percentage distribution of each active ingredient and AMPA in the soil and water of the wetland is reported.

As clearly shown in Figure 3, the pesticides were found not homogeneously distributed in the SFCW water at the five sampling positions. As expected, the water volume pumped inside the system during the pesticide distribution was enough to move most part of the pesticides into the second half of the system without enabling them to reach the end point (position 5). Their absence at position 5 showed that, unless in the case of subsequent extreme rain events, the real scale SFCW can provide good conditions and residence time needed for an effective retention of agricultural pollutants.

As far as the monitoring points 1–4 are concerned, each substance was found at quite similar concentrations at positions 1–3 and at the highest value at position 4. The high spatial variability of pesticide distribution indicated that the pesticides were not distributed uniformly within the first three meanders, likely due to the complex conditions of the system and numerous possible factors (e.g., different roughness, slope, infiltration, vegetation density) affecting the water flow. In addition, for imidacloprid and dimethomorph, a general increase of the concentrations was observed in water from days 1 to 7 at positions 1–3, noticeably at position 1. A high heterogeneity of pesticide concentrations in soil and water samples at a few days after treatment, as well as an increase in the concentration from the first to the second samples after treatment, is very common phenomena in field studies (EU DG-SANCO, 2006). In our study, from days 1 to 7, an increase in the imidacloprid and dimethomorph concentrations at positions 1–3 and a backward diffusion from positions 4 to 1 occurred due to the absence of water inflow pumping. For the same period, the fast dissipation of glyphosate dominated any possible diffusion phenomena.

As far as the SFCW soil is concerned, imidacloprid and dimethomorph were more homogeneously distributed among positions 1–4 than what was observed in water samples, and almost absent at position 5. Differently, glyphosate did not show detectable concentration at any sampling position and for the entire duration of the trial, except for traces (0.06%) at day 21 and position 1. This observation could be explained by the main mechanisms for glyphosate removal in aquatic environments through the formation of stable complexes with metals such as Fe, Al, and Cu occurring on the surfaces of inorganic and organic components of soils and sediments (Liu et al., 2019; Bento et al. 2018).

Under field conditions, the pesticide dissipation is influenced by many environmental factors, which are spatially variable at a small scale (EU DG-SANCO, 2006). To average the spatial variability and to describe the dissipation potential of the wetland as a whole, in Figure 4, the concentration (mg kg^−1^) of each pesticide and AMPA in the water and soil compartments (Figure 4A) and the percentage of the initially applied amount (Figure 4B) were reported as a mean of the 1–5 positions with time.

Notably, as shown in Figure 4B, already after 1 week from the application, about 50%, on average, of the initially applied dose of each pesticide was dissipated by the wetland. After 8 weeks from the application, the soil-water system contained ca. 12% of initially applied imidacloprid, 4% of dimethomorph, and traces of AMPA.

Among the pesticides, some differences in their distribution between the soil and water phases (Figure 4B) could be related to their chemical and physical characteristics. The occurrence of high percentages of the initially applied amount of cationic imidacloprid in the water phase and low accumulation in the soil for the entire period of observation was related to its high hydrophilic nature (Sw = 0.610 g L^−1^, logKow = 0.57). The more hydrophobic character of dimethomorph (Sw = 0.285 g L^−1^; logKow = 2.68) caused higher pesticide accumulation in the soil and lower occurrence in the water than what was observed for imidacloprid. Finally, despite its high solubility (Sw = 10.5 g L^−1^), glyphosate was detected in the SFCW water only during the first week and at the lowest percentage level if compared with the other pesticides. Highly soluble AMPA (Sw = 1467 g L^−1^), which is formed by glyphosate microbial transformation, was detected only in water from week 2 until the end of the trial. Glyphosate and AMPA were not found in the SFCW soil owing to their known irreversible reactions with soil components (Maqueda et al. 2017; Bois et al. 2013; Farenhorst et al. 2009).

### Dissipation kinetics of the pesticides within the SFCW

The average concentrations of pesticides among 1–5 sampling positions, reported in Figure 4A, are used for modeling their dissipation in the SFCW water phase and in the soil-water system. In Table 3, the first-order (FO) fittings for the dissipation of three pesticides, both in the water phase and in the water-soil system, were optimized by including the initial theoretical concentration and excluding the data at day 1. For the dissipation of AMPA, following the indications of EU DG-SANCO (2006) for metabolites, the fitting was calculated from its highest concentration value that was observed at day 21.

**Table 3:** Dissipation kinetics of the selected pesticides in the SFCW water phase and water-soil system. Calculated half-life time (DT_50_) and time to reduce 90% of initially added amount of active ingredients (DT_90_), if available, are reported

Dissipation kinetics*	**Imidacloprid**	**Dimethomorph**	**Glyphosate**	**AMPA**
*Water phase*				
**1**^**st**^ **order:** ln[*C*]_0_/[*C*]_*t*_ = *kt*				
**Kinetic equation**	*y* = 0.033*x* + 0.4094	*y* = 0.0578*x* + 0.4454	*y* = 0.1196*x* + 1.9612	*y* = 0.0441*x*−0.9005
***R***^**2**^	0.8545	0.9265	0.8166	**0.9868**
**DT**_**50**_ (days)	20.6	12.0	5.8	36.7
**DT**_**90**_ (days)	68.5	39.8	19.3	73.2
**Hockey-stick model:** (1) ln[*C*]_0_/[*C*]_*t*_ = *k*_1_*t* for *t* ≤ 28 days				
**Kinetic equation**	*y* = 0.0629*x* + 0.0696	*y* = 0.0879*x* + 0.0775	*y* = 0.2318*x* + 0.6152	-
***R***^**2**^	**0.9811**	**0.9742**	**0.9620**	-
**DT**_**50**_ (days)	11.1	7.9	3.0	-
(2) ln[*C*]_0_/[*C*]_*t*_ = *k*_2_*t* for *t* > 28 days				
**Kinetic equation**	*y* = 0.0205*x*−0.7453	*y* = 0.0323*x*−1.162	-	-
***R***^**2**^	0.9401	0.8606	-	-
*Soil-water phase*				
**1**^**st**^ **order:** *l*ln[*C*]_0_/[*C*]_*t*_ = *kt*				
**Kinetic equation**	*y* = 0.0289*x* + 0.3544	*y* = 0.0518*x* + 0.3233	*y* = 0.1201*x* + 1.9215	*y* = 0.0441*x*−0.9005
***R***^**2**^	0.7787	0.9167	0.8238	**0.9868**
**DT**_**50**_ (days)	24.0	13.4	5.8	36.7
**DT**_**90**_ (days)	79.7	44.5	19.2	73.2
**Hockey-stick model:** (1) ln[*C*]_0_/[*C*]_*t*_ = *k*_1_*t* for *t* ≤ 28 days				
**Kinetic equation**	*y* = 0.0626*x*−0.0238	*y* = 0.0853*x*−0.0575	*y* = 0.02286*x* + 0.6152	-
***R***^**2**^	**0.9938**	**0.9880**	**0.9582**	-
**DT**_**50**_ (days)	11.1	8.1	8.1	-
(2) ln[*C*]_0_/[*C*]_*t*_ = *k*_2_*t* for *t* > 28 days				
**Kinetic equation**	*y* = 0.0218*x*−0.7025	*y* = 0.0415*x*−1.4078	-	-
***R***^**2**^	**0.8858**	**0.9786**	-	-

*Best fittings obtained including the theoretical initial concentration and excluding the concentration at day 1; *y* = concentration (mg L^−1^ or mg kg^−1^); *x* = *t* (days); breakpoint of the Hockey-stick model = 28 days. AMPA kinetics calculated from maximum concentration (day 21) to day 56

**Figure 5:**
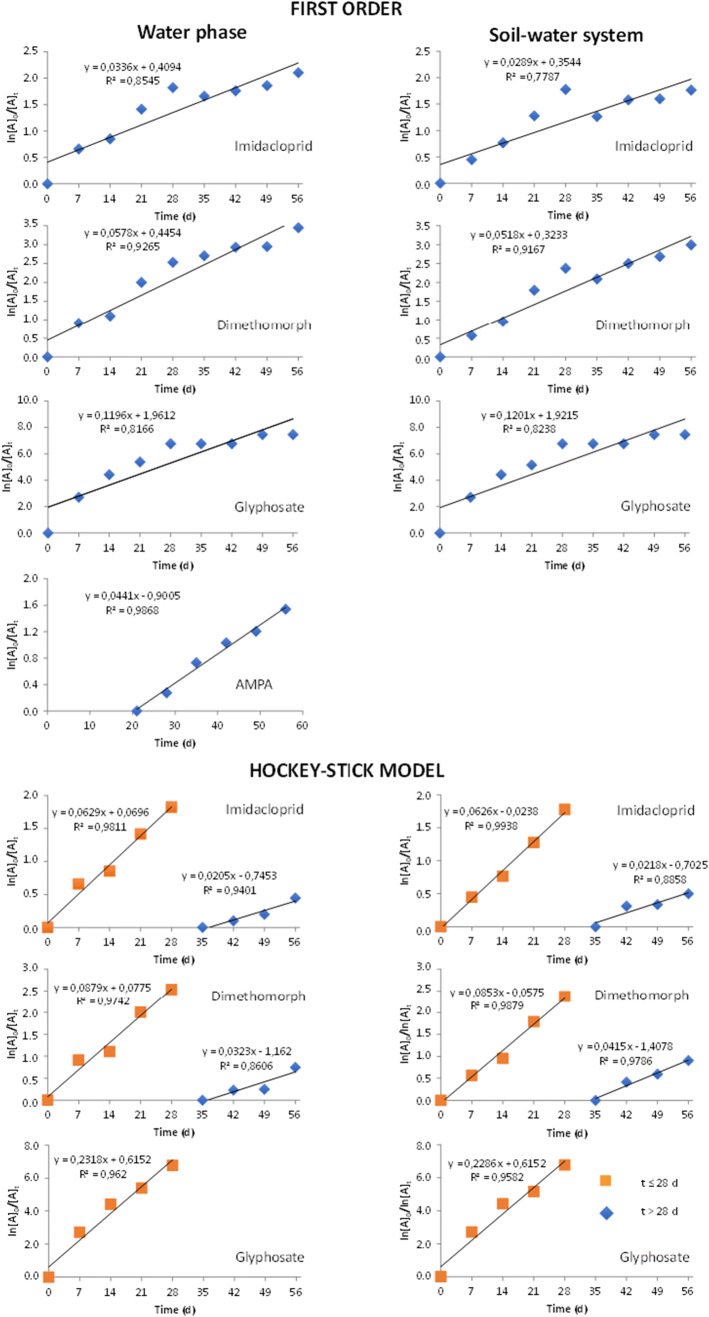
Regression lines for first-order and Hockey-stick fittings (breakpoint at 28 days) of the pesticide dissipation in the SFCW water phase and soil-water system

Acceptable FO fittings were obtained in both the water and soil-water phases for imidacloprid (*r*^2^ = 0.8545 and 0.7787, respectively), dimethomorph (*r*^2^ = 0.9265 and 0.9167), glyphosate (*r*^2^ = 0.8166 and 0.8238), and AMPA (*r*^2^ = 0.9868). The calculated FO DT_50_ in the water and in the water-soil system (21–24 days, 12–13 days, 5.8–5.8 days, and 37 days for imidacloprid, dimethomorph, glyphosate, and AMPA, respectively) largely overcome the experimental values (experimental DT_50_ of ca. 7 days for imidacloprid and dimethomorph, <1 day for glyphosate, and ca. 14 days for AMPA). The regression curves for the FO fittings are reported in Figure 5.

As the initial fast decrease in pesticide concentrations up to 28 days was clearly followed by a slower decline (Figure 4A), the pesticide concentrations were also fitted to the bi-phase Hockey-stick (HS) model with breakpoint at day 28. Again, the best HS fitting was obtained by including the initial concentration and excluding the data at day 1. In general, the model better described the dissipation of the three pesticides before the breakpoint both in the water phase and in the soil-water system (*r*^2^ = 0.9811 and 0.9938; 0.9742 and 0.9880; 0.9620 and 0.9582 for imidacloprid, dimethomorph, and glyphosate, respectively) than the FO model. The calculated HS DT_50_ for imidacloprid and dimethomorph in the water phase and soil-water system (11.1–11.1 and 7.9–8.1 days, respectively) were shorter than the FO DT_50_ values and closer to the experimental half-life times. As far as glyphosate was concerned, the HS DT50 (3.0 days) in the water was more in line with the experimental one (<1 day), whereas the DT_50_ in soil-water system was certainly overestimated by its unique detection in the soil at 21 days. The regression curves for the HS fittings, before and after the breakpoint, are reported in Figure 5. After the breakpoint, acceptable fittings were observed in the water phase and in the soil-water system for imidacloprid (*r*^2^ = 0.9401and 0.8858) and dimethomorph (*r*^2^ = 0.8606 and 0.9786). On the contrary, the quite constant concentration values of glyphosate close to the LOD were not suitable to be fitted.

Although the HS model better described the pesticide dissipation kinetics, the calculated FO DT_50_ values were considered in the following for a proper comparison with the DT_50_ available in the literature where FO modeling is generally adopted.

Among pesticides in our system, imidacloprid was the most recalcitrant pesticide to dissipate. Comparing the FO DT_50_ of imidacloprid in our wetland water-soil system and water phase (24.0 and 20.6 days, respectively, Table 3) with the average DT_50_ reported on the Pesticide Properties Database (PPDB) (Lewis et al. 2016) for the water-sediment system in the absence of plants and the relative water phase (129 and 30 days, respectively, Table 1), it was possible to gather how the specific vegetated wetland environment in the study was able to speed up the pesticide dissipation.

With respect to dimethomorph, the DT_50_ calculated in our soil-water system and water phase (13.4 and 12.0 days, respectively, Table 3) was in line with the average value reported by the PPDB (Lewis et al. 2016) for the water-sediment system and the relative water phase (38 and 10 days, respectively, Table 1).

As far as glyphosate was concerned, its dissipation in the wetland (DT_50_ in both water-soil system and water phase of 5.8 days, Table 3) was also in line with the average DT50 of 9.9 days reported by PPDB for the water phase in contact to sediment (Table 1). Conversely, the higher average DT_50_ value (74.5 days) reported by the PPDB for the water-sediment system was not observed in our wetland.

Microbial transformations of glyphosate to AMPA and irreversible reactions with metals anchored to soil surfaces (Maqueda et al. 2017; Farenhorst et al. 2009) and sediment components (Liu et al., 2019; Bento et al. 2018) are considered the main mechanisms for its removal in aquatic environments. On the contrary, hydrolytic and photolytic transformations of glyphosate can be considered negligible (NPIC, 2011). In our system, the fast glyphosate removal (approx. 50% of the initially added amount) from the SFCW water already after 1 h of contact, as assessed during the evaluation of extraction recovery efficiency (see Supplementary Material), was in line with a study on glyphosate mitigation in microcosms simulating stormwater basins (Bois et al. 2013) whose characteristics (sediment mix with 19% of carbonates and water pH of 8) were likely those of our SFCW. Similarly to our findings, in Bois et al. (2013), an efficient adsorption of the herbicide at initial concentration of 50 mg L^−1^ by sediment was achieved within 6 h; after that, its biodegradation lasted 5 weeks.

### Behavior of glyphosate in the SFCW

The formation of insoluble complexes of glyphosate through binding to Fe/Al-OH_*x*_ groups on the surfaces of soil components (Maqueda et al. 2017; Farenhorst et al. 2009), with Ca, Mg, Cu, and Mn in water at pH 7 and in different soils buffered at the same pH has been widely assessed (i.e., Sundaram and Sundaram, 1997). In our study, the mechanism of glyphosate binding to the solid components of the wetland soil could not be investigated by IR analysis owing to the overlapping of vibrational bands of the herbicide with those of the soil matrix (Figure SM.3 and its description as a Supplementary Material).

As already mentioned, despite the fast dissipation of glyphosate, traces of the herbicide and small amounts of AMPA were revealed in the water phase until the end of the 8-week trial. The ability of cations occurring in the wetland water phase at relevant concentrations (namely, Ca = 56.4 mg L^−1^, Mg = 15.1 mg L^−1^, Na = 10.1 mg L^−1^, and Cu = 0.178 mg L^−1^, Table SM.2) to complex glyphosate was supposed supporting this observation.

It is of general knowledge that glyphosate, in the form of highly soluble zwitterionic species bearing two net negative charges (namely, ^−^OOC-CH_2_-NH_2_^+^-CH_2_-PO_3_^2−^) at the alkaline pH of the wetland water (pH = 8), is suitable to complex cations. There are plenty of model studies that have already reported on the formation and stability of soluble complexes of glyphosate with transition metal and divalent alkaline earth cations in synthetic water (Undabeytia et al. 2002; Abate et al. 1996; Daniele et al. 1997). The formation and stability constants of soluble complexes of glyphosate with metal cations as Ca, Mg, and Cu in water within a wide range of pH and ionic strength values has been deeply investigated (Abate et al. 1996; Daniele et al. 1997). However, studies dealing with the formation and observation of complex between glyphosate and cations contained into natural waters are not available, owing to the complexity of these media.

**Figure 6:**
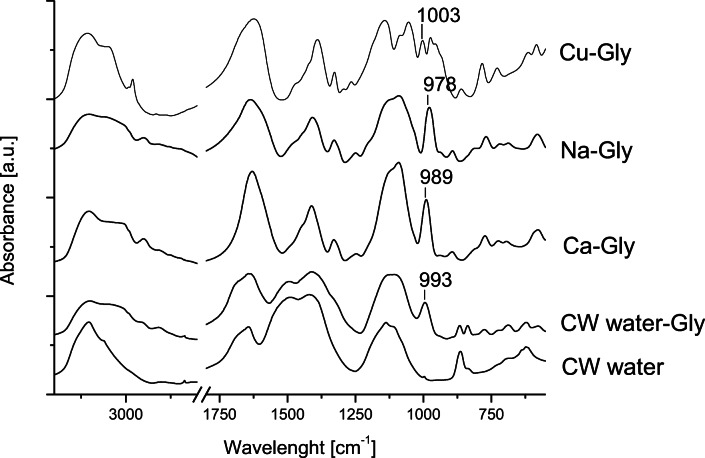
FT-IR spectra of the following: (i) freeze-dried CW water solutes (CW water), of glyphosate added to the SFCW water (CW water-Gly), and of glyphosate added to synthetic water at pH 8 containing Ca^2+^, Na^+^, and Cu^2+^ at the eq_(+)_:eq_(−)_ ratio of 1:1 (Ca-Gly, Na-Gly, and Cu-Gly, respectively)

Here, the possible formation of soluble complexes between glyphosate and the cations occurring in the SFCW water phase was investigated by comparing the IR spectra of freeze-dried samples of the wetland water in the presence or in the absence of glyphosate (Figure 6). The spectrum of the water solutes in the absence of glyphosate (CW water spectrum) showed absorbances typical of dissolved organic matter and hydration water of ionic species (Oren and Chefetz, 2012; Barber et al. 2001). In the presence of glyphosate (CW water-Gly spectrum), the spectrum was deeply modified. Additional bands could be observed in the 3800–2700-cm^−1^ region. In addition, the bands centered at 1643 and 1410 cm^−1^ enhanced their intensity, and a new band at 993 cm^−1^ appeared.

To better define the contribution of the most representative cations of wetland water phase to the soluble forms of glyphosate in the CW water-Gly spectrum, spectra of glyphosate complexed to Ca, Na, and Cu in synthetic water at pH 8 were produced as models (namely, Ca-Gly, Na-Gly, and Cu-Gly spectrum, respectively). The assignment of the most relevant broad and sharp bands of Na-Gly, Ca-Gly, and Cu-Gly model spectra are reported in Table 4. A more detailed interpretation of the spectrum of Cu-glyphosate complex can be found in Undabeytia et al. (2002).

**Table 4:** Assignments of main infrared absorbances in the 2000–600-cm^−1^ spectral range of complexes of glyphosate with Na^+^, Ca^2+^, and Cu^2+^ ions at pH 8. Glyphosate structure at pH 8: ^−^OOC-CH_2_-NH_2_^+^-CH_2_-PO_3_^2−^

**Vibrational mode**	**Na-Gly complex**^(1)^	**Ca-Gly complex**	**Cu-Gly complex**^(2)^
-COO^(−)^*ν*_*asym*_*;* -NH_3_^+^*def;* -OHδ	1634 (broad)	1630 (broad)	1622 (broad)
-COO^(−)^*ν*_*sym*_*;* -NH_3_^+^*def,* -CH_2_-*def*	1408 (broad)	1412 (broad)	1391 (broad)
-CH_2_- *def*	1329	1329	1327
-PO_3_^2-^ *ν*	1090 (broad)	1090 (broad)	1142; 1055
-CH_2_- *def*	978 (sharp)	989 (sharp)	1003
-PO_3_^2-^ *ν*			974; 955

^(1)^Miano et al. 1992; ^(2)^ Undabeytia et al. 2002

Interesting to note, the complexation between the heavy phosphonate moiety and the single cations sensibly affected the deformation mode of the phosphonate adjacent -CH_2_- group that was found at different positions in the model spectra of Na-Gly, Ca-Gly, and Cu-Gly (978, 989, and 1003 cm^−1^, respectively, Figure 6). This band was thus adopted as a marker for the identification of specific complexes between glyphosate and the single cations in the wetland water phase.

In CW water-Gly spectrum (Figure 6), although the overlapping of the glyphosate bands with those of organic and inorganic solutes of the wetland water did not allow direct observation of specific interactions between the herbicide -PO_3_^2−^ group and the cations occurring in the wetland water, the deformation mode of glyphosate -CH_2_- groups was clearly identifiable at 993 cm^−1^ (Table 4).

The presence of this band at 993 cm^−1^ in the CW water-Gly spectrum confirmed the main interaction of calcium with the herbicide in the wetland water phase and confirmed this band as a marker of the interaction of glyphosate with calcium ion in natural water media.

Any metal cation, whether adsorbed on soil components or in soil solution, able to strongly complex glyphosate can affect the dynamics of adsorption or desorption of the herbicide by soils (Morillo et al. 2000). In our SFCW, made of calcareous soil and water phase with relevant concentration of calcium, the glyphosate solubilization seemed mostly driven by calcium ions.

These findings can be of relevant interest for aquatic systems occurring on calcareous soils of Mediterranean areas. It is reasonable to hypothesize that, in the form of stable soluble complex with calcium ion, traces of glyphosate can be protected by irreversible reactions with the soil solid components. At the same time, the complexed herbicide, which is kept in solution, is available to biotic transformations as indicated by the formation and occurrence of its main metabolite AMPA from the second week until the end of the monitoring period.

## Conclusions

As a general conclusion, the observed dissipation profile of the three pesticides showed that the specific vegetated wetland can treat a mixture of pesticides at very high concentrations. Considering the most persistent pesticides dimethomorph and imidacloprid, a prolonged hydraulic residence time in the SFCW system is an important variable to depollute the waters, preventing hazards to other aquatic ecosystems. The results obtained are particularly relevant as they depict the behavior of a full-scale surface flow constructed wetland, which is difficult to control for its own nature, under real operational conditions. Based on the results obtained, constructed wetlands seem an effective technology, able to play a key role in the sustainability of agriculture.

## Supplementary information

ESM 1 (DOCX 463 kb)

## Data Availability

Not applicable.
