# Supplementary material for: Removal and fate of pesticides in a farm constructed wetland for agricultural drainage water treatment under Mediterranean conditions (Italy)
Source: Environ Sci Pollut Res Int. 2021 Sep 2;29(5):7283–99. doi: 10.1007/s11356-021-16033-4 (PMC8763787; doi:10.1007/s11356-021-16033-4)
Supplement: Supplementary file 1 — (DOCX 463 kb) [file 11356_2021_16033_MOESM1_ESM.docx]

**Removal and fate of pesticides in a farm constructed wetland for agricultural drainage water treatment under Mediterranean conditions (Italy)**

**-Supplementary MATERIAL-**

Ilaria Braschi ^1,2^, Sonia Blasioli ^1*^, Stevo Lavrnić ^1^, Enrico Buscaroli ^1^, Katia Di Prodi ^2,3^, Domenico Solimando ^4^, Attilio Toscano ^1^

^1^ Department of Agricultural and Food Sciences, University of Bologna, 40127 Bologna, Italy

^2^ GRIFA Gruppo di Ricerca Italiano Fitofarmaci e Ambiente, 09124 Cagliari, Italy

^3^ Central Laboratory of Conserve Italia Group, Conserve Italia Soc. Coop. Agricola, 40068 San Lazzaro di Savena (BO) Italy

^4^ Consorzio di Bonifica Canale Emiliano Romagnolo, 40137 Bologna, Italy

**Corresponding author. E-mail address:* [*sonia.blasioli@unibo.it*](mailto:sonia.blasioli@unibo.it)

***SM.1 Recovery efficiency of pesticide extraction from water and soil samples***

An assessment of the recovery efficiency of the three pesticides from the SFCW soil-water system was performed before pesticide distribution. Briefly, a fresh soil-water sample was collected from the wetland and placed into a 100 mL plastic beaker. The aqueous solution was manually separated from the soil and spiked with a mixture of the three pesticide active substances (PESTANAL, analytical standards, Merk) at about 100 and 10% of the initially applied dose (namely, 1.0 and 0.1 mg L^-1^ for imidacloprid or dimethomorph and 1.5 and 0.15 mg L^-1^ for glyphosate). After complete pesticide dissolution by mixing/sonication cycles, the aqueous solution was placed in contact with the soil phase. After 60 minutes of contact time, the water phase was separated from the soil by centrifugation and the solid and liquid phases were processed as described above.

For imidacloprid, the recovery efficiency from the SFCW water-soil system was of 96.5±0.6 and 95.1±1.0% at 100 and 10% of initially applied dose, respectively (namely, 77.0±0.5 and 74.2±0.7% from the water at 100 and 10% of applied dose, respectively, and 19.5±0.1 and 20.9±0.3% from the soil at 100 and 10% iad, respectively). For dimethomorph, the recovery efficiency resulted 98.0±7.0 and 96.9±8.8% at 100 and 10% of applied dose, respectively (namely, 55.0±5.0% and 56.1±5.3% from the water at 100 and 10% of applied dose, respectively, and 43.0±2.0 and 40.8±3.5% from the soil at 100 and 10% of applied dose, respectively). For glyphosate, the recovery efficiency was 52.2±2.0 and 45.0±5.1% at 100 and 10% of applied dose, respectively (namely, 49.3±1.7 and 45.0±5.1% from the water at 100 and 10% of applied dose, respectively, and 2.9±0.3% from soil at only 100% of applied dose).

***SM.2 HPLC-MS/MS conditions for pesticide analysis***

For the analysis of imidacloprid and dimethomorph, the HPLC system bore a Hypersil GOLD™ column (C18, 50×2.1mm×1.9 μm) kept at 40°C. The analysis was operated at the eluant flow of 0.4 mL min^-1^ under gradient condition. The mobile phase was composed by a mixture of (A) 5 mM ammonium formiate + 0.05% formic acid in LC-MS grade H_2_O (Merck Millipore) and (B) 5 mM ammonium formiate and 0.05% formic acid in LC-MS grade methanol (Merck Millipore). The gradient conditions were: 0-2 min 60% (A) + 40% (B), 2-6 min 50% (A) + 50% (B), 6-8 min15% (A) + 85% (B), 8-9 min 15% (A) + 85% (B), 9-13 min 5% (A) + 95% (B), 13.0-13.5 min 60% (A) + 40% (B,) and from 13.5-17.8 min 60% (A) + 40% (B). In these conditions, the retention time of imidacloprid and dimethomorph was of 0.69 and 5.29 min, respectively. The ESI capillary voltage was set to 4000 in positive polarity mode and the MS/MS analyser in Multiple Reaction Mode. Precursor ions for dimethomorph and imidacloprid were 388.1 and 256.0 *m/z*, respectively. Product ions for quantitation and confirmation were 301.1 and 165.1 *m/z,* respectively, for dimethomorph and 208.9 and 175.0 *m/z* for imidacloprid. The limit of quantitation (LOQ) and the limit of detection (LOD) for both the pesticides in soil samples were 0.05 and 0.005 mg kg^-1^, respectively, whereas in water samples they were 0.05 and 0.008 mg L^-1^, respectively.

For glyphosate and AMPA LC-MS/MS analysis, the system bore a Hypercarb™ (PGC, 100×2,1mm × 5 μm) kept at 40°C under gradient condition and variable flow rate. The eluant was composed by a mixture of (A) 1% acetic acid + 5% LC-MS grade methanol (Merck Millipore) in LC-MS grade H_2_O (Merck Millipore), and (B) 1% acetic acid in LC-MS grade methanol. The gradient conditions were: 0 min 100% (A) (flow at 0.4 mL min^-1^), 0-10 min (A) from 100% to 10% (flow from 0.4 to 0.5 mL min^-1^); 10-40 min 10% (A) + 90% (B); 40-50 min (A) from 10% to 100% (flow at 0.4 mL min^-1^). In these conditions, the retention time of glyphosate and AMPA were 0.90 and 0.83 min, respectively. The ESI voltage was set to 4500 in negative polarity mode and the MS/MS analyser in MRM. Precursor ions for glyphosate and AMPA were 169.9 and 110.0 *m/z*. Product ions for quantitation and confirmation were 62.9 and 150.0 *m/z*, respectively, for glyphosate, and 63.0 and 79.0 *m/z* for AMPA. The limit of quantitation (LOQ) and the limit of detection (LOD) for both glyphosate and AMPA in soil samples were 0.05 and 0.008 mg kg^-1^, respectively, whereas in water samples they were 0.05 and 0.005 mg L^-1^, respectively.

**Table SM.1** Characterization of the SFCW water. The analyses were performed in triplicate.

| **Parameter** | **Unit** | **Value** | **St. dev.** |
| --- | --- | --- | --- |
| Chemical oxygen demand | mg L^-1^ | 7.72 | ±1.20 |
| Biochemical oxygen demand | mg L^-1^ | 2.00 | ±0.12 |
| Total suspended solids | mg L^-1^ | 34.30 | ±0.88 |
| Total dissolved solids | mg L^-1^ | 290.00 | ±7.00 |
| Turbidity | NTU | 24.40 | ±2.12 |
| Total organic carbon | mg L^-1^ | 15.70 | ±0.08 |
| Dissolved oxygen | mg L^-1^ | 4.07 | ±0.10 |
| Dissolved oxygen | % saturation | 48.40 | ±1.19 |
| Electrical conductivity | mS cm^-1^ | 0.43 | ±0.02 |
| Water hardness | dH | 10.70 | ±0.65 |
| Redox potential | mV | 156.10 | ±1.15 |
| pH |  | 7.96 | ±0.02 |
| Temperature | °C | 25.50 | ±0.01 |
| Total nitrogen | mg L^-1^ | 2.31 | ±0.15 |
| Ammonium-nitrogen | mg L^-1^ | 0.215 | ±0.004 |
| Nitrate-nitrogen | mg L^-1^ | 1.690 | ±0.012 |
| Total phosphorus | mg L^-1^ | 0.068 | ±0.006 |
| Phosphate-phosphorus | mg L^-1^ | 0.029 | ±0.002 |
| Aluminum | mg L^-1^ | 0.010 | ±0.005 |
| Boron | mg L^-1^ | 0.016 | ±0.010 |
| Calcium | mg L^-1^ | 56.40 | ±0.09 |
| Chromium | mg L^-1^ | 0.038 | ±0.003 |
| Chromium(VI) | mg L^-1^ | 0.027 | ±0.003 |
| Copper | mg L^-1^ | 0.178 | ±0.017 |
| Iron | mg L^-1^ | 0.109 | ±0.012 |
| Lead | mg L^-1^ | 0.110 | ±0.011 |
| Magnesium | mg L^-1^ | 15.1 | ±0.1 |
| Manganese | mg L^-1^ | 0.016 | ±0.004 |
| Molybdenum | mg L^-1^ | 1.57 | ±0.08 |
| Nickel | mg L^-1^ | 0.066 | ±0.017 |
| Sodium | mg L^-1^ | 10.11 | ±0.09 |
| Tin | mg L^-1^ | 0.194 | ±0.009 |
| Zinc | mg L^-1^ | 0.022 | ±0.006 |
| Cyanide | mg L^-1^ | 0.008 | ±0.002 |
| Chloride | mg L^-1^ | 22.20 | ±0.32 |
| Phenols | mg L^-1^ | 0.06 | ±0.01 |
| Sulfate | mg L^-1^ | 37.3 | ±0.7 |
| Total surfactants | mg L^-1^ | 0.333 | ±0.042 |
| Ionic surfactants | mg L^-1^ | 0.214 | ±0.050 |
| Non-ionic surfactants | mg L^-1^ | 0.13 | ±0.02 |
| Total coliforms | CFU 100 mL^-1^ | 17,000 | ±1000 |
| *Escherichia coli* | CFU 100 mL^-1^ | 40 | ±1 |

**Table SM.2** The main physico-chemical characteristics of the SFCW soil up to 60 cm of depth. In brackets relative errors are expressed as relative standard deviation.

| Sampling Point | 1 | | | | | 2 | | | | | 3 | | | | | 4 | | | | | |
| --- | --- | --- | --- | --- | --- | --- | --- | --- | --- | --- | --- | --- | --- | --- | --- | --- | --- | --- | --- | --- | --- |
| Sampling Depth [cm] | 0-5 | 5-15 | 15-30 | 30-45 | 45-60 | 0-5 | 5-15 | 15-30 | 30-45 | 45-60 | 0-5 | 5-15 | 15-30 | 30-45 | 45-60 | 0-5 | 5-15 | 15-30 | 30-45 | 45-60 |  |
| Parameter [unit] |  |  |  |  |  |  |  |  |  |  |  |  |  |  |  |  |  |  |  |  |  |
| Humidity [g kg^-1^] | n.a. | 213.7 | 201.0 | 184.0 | 190.2 | n.a. | 225.4 | 190.4 | 187.3 | n.a. | n.a. | 234.6 | 200.3 | 174.2 | 181.4 | n.a. | 193.7 | 193.1 | 205.0 | 222.9 |  |
| Bulk density [g cm^-3^] | n.a. | 1.9 | 2.1 | .21 | 2.1 | n.a. | 2.1 | 2.0 | 2.2 | n.a. | n.a. | 2.1 | 2.1 | 2.3 | 2.2 | n.a. | 2.3 | 2.1 | 2.2 | 2.2 |  |
| Sand content [g kg^-1^] | 115.0 (0.1) | 79.0 (1.1) | 80.0 (1.0) | 74.0 (1.4) | 71.0 (4.0) | 159.0 (0.1) | 125.0 (1.5) | 96.0 (1.5) | 87.0 (3.2) | 77.0 (10.1) | 134.0 (0.1) | 108.0 (5.7) | 102.0 (3.3) | 105.0 (1.2) | 191.0 (1.9) | 148.0 (0.1) | 95.0 (0.6) | 86.0 (1.2) | 47.0 (1.9) | 32.0 (2.2) |  |
| Silt content [g kg^-1^] | 610.0 (0.1) | 605.0 (3.1) | 617.0 (6.4) | 609.0 (3.0) | 613.0 (3.6) | 429.0 (0.1) | 463.0 (8.8) | 450.0 (12.7) | 542.0 (4.1) | 579.0 (11.4) | 536.0 (0.1) | 562.0 (8.0) | 582.0 (3.9) | 607.0 (9.8) | 630.0 (3.7) | 577.0 (0.1) | 575.0 (6.9) | 653.0 (8.8) | 720.0 (8.2) | 747.0 (0.1) |  |
| Clay content [g kg^-1^] | 275.0 (0.1) | 316.0 (6.1) | 302.0 (12.8) | 316.0 (6.1) | 316.0 (6.2) | 413.0 (0.1) | 413.0 (9.4) | 454.0 (12.9) | 371.0 (5.2) | 344.0 (17.0) | 330.0 (0.1) | 330.0 (11.8) | 316.0 (6.2) | 289.0 (20.2) | 179.0 (10.9) | 275.0 (0.1) | 330.0 (11.8) | 261.0 (22.3) | 234.0 (25.0) | 220.0 (0.1) |  |
| pH (H_2_O) | 8.3 (0.1) | 8.5 (0.1) | 8.6 (0.3) | 8.6 (0.3) | 8.7 (1.9) | 8.2 (0.2) | 8.3 (0.5) | 8.7 (0.3) | 8.7 (0.2) | 8.6 (1.3) | 8.4 (0.1) | 8.5 (0.2) | 8.7 (0.3) | 8.7 (0.2) | 8.7 (0.1) | 8.2 (0.6) | 8.4 (0.4) | 8.5 (0.9) | 8.6 (0.5) | 8.6 (1.1) |  |
| pH (CaCl_2_) | 7.8 (0.4) | 7.9 (0.3) | 7.9 (0.2) | 7.9 (0.9) | 7.9 (0.1) | 7.8 (0.7) | 7.8 (0.5) | 8.0 (0.1) | 8.1 (0.9) | 8.1 (0.1) | 8.1 (0.2) | 8.0 (0.1) | 8.0 (0.6) | 8.1 (0.1) | 8.1 (0.2) | 7.6 (0.1) | 8.0 (0.1) | 8.0 (0.3) | 8.1 (0.3) | 8.1 (0.4) |  |
| Electric conductivity [μS cm^-1^] | 606.9 (12.3) | 353.1 (0.1) | 331.0 (1.2) | 387.3 (1.2) | 456.2 (2.5) | 593.8 (9.4) | 464.6 (11.9) | 344.4 (9.3) | 345.4 (4.7) | 399.2 (10.5) | 510.8 (3.3) | 404.0 (1.7) | 362.7 (2.0) | 362.9 (4.2) | 388.3 (0.6) | 486.7 (1.9) | 377.5 (0.1) | 363.7 (0.3) | 342.3 (2.1) | 350.3 (3.9) |  |
| Total carbonates [g kg^-1^] | 158.0 (1.8) | 151.0 (1.4) | 142.0 (1.8) | 148.0 (0.6) | 155.0 (11.1) | 158.0 (5.2) | 148.0 (2.1) | 155.0 (4.6) | 169.0 (0.3) | 221.0 (0.6) | 138.0 (5.0) | 145.0 (0.4) | 137.0 (3.8) | 134.0 (2.5) | 156.0 (2.3) | 120.0 (4.7) | 123.0 (6.3) | 124.0 (2.5) | 161.0 (6.6) | 193.0 (2.0) |  |
| Total organic carbon [g kg^-1^] | 36.0 (3.1) | 13.0 (8.5) | 11.0 (0.7) | 11.0 (1.6) | 11.0 (7.6) | 46.0 (22.8) | 18.0 (17.8) | 8.9 (2.7) | 6.3 (3.0) | 5.0 (5.0) | 28.0 (10.7) | 20.0 (17.0) | 10.0 (7.5) | 9.3 (1.4) | 6.9 (1.4) | 56.0 (13.9) | 14.0 (11.2) | 11.0 (4.7) | 6.8 (2.5) | 5.8 (5.8) |  |
| Total nitrogen [g kg^-1^] | 3.5 (2.6) | 1.4 (1.4) | 1.3 (0.7) | 1.3 (1.3) | 1.3 (1.3) | 3.9 (3.9) | 1.8 (1.8) | 1.1 (0.8) | 0.77 (0.77) | 0.6 (0.6) | 2.4 (2.4) | 1.8 (1.8) | 1.2 (1.2) | 1.2 (1.2) | 0.85 (0.85) | 4.3 (4.3) | 1.5 (1.5) | 1.3 (0.6) | 0.84 (0.84) | 0.68 (0.68) |  |
| Carbon/nitrogen ratio | 10.4 | 9.1 | 8.4 | 8.5 | 8.4 | 11.9 | 9.8 | 8.3 | 8.2 | 8.2 | 11.7 | 10.9 | 8.4 | 7.9 | 8.2 | 13.0 | 9.3 | 8.2 | 8.1 | 8.6 |  |
| As [mg kg^-1^] | 5.1 (5.1) | 5.8 (4.3) | 5.6 (5.6) | 5.5 (5.5) | 5.9 (4.7) | 4.3 (4.3) | 5.5 (3.3) | 5.3 (5.6) | 5.0 (6.3) | 5.2 (3.6) | 5.4 (3.6) | 5.8 (2.9) | 5.6 (3.4) | 5.2 (3.3) | 5.2 (4.3) | 4.5 (3.7) | 5.6 (2.5) | 5.4 (4.3) | 5.2 (5.2) | 5.0 (3.3) |  |
| Be [mg kg^-1^] | 1.2 (1.2) | 1.3 (1.3) | 1.3 (1.3) | 1.3 (1.3) | 1.3 (1.3) | 1.2 (1.2) | 1.3 (1.3) | 1.2 (1.2) | 1.2 (1.2) | 1.1 (1.1) | 1.3 (1.3) | 1.3 (1.3) | 1.3 (1.3) | 1.2 (1.2) | 1.1 (1.1) | 1.2 (1.2) | 1.3 (1.3) | 1.3 (1.3) | 1.3 (1.3) | 1.2 (1.2) |  |
| Cd [mg kg^-1^] | 0.2 (0.2) | 0.2 (0.2) | 0.1 (0.1) | 0.2 (0.2) | 0.2 (0.2) | 0.2 (0.2) | 0.1 (0.1) | 0.1 (0.1) | 0.1 (0.1) | 0.1 (0.1) | 0.2 (0.2) | 0.1 (0.1) | 0.1 (0.1) | 0.1 (0.1) | 0.1 (0.1) | 0.2 (0.2) | 0.2 (0.2) | 0.2 (0.2) | 0.2 (0.2) | 0.1 (0.1) |  |
| Co [mg kg^-1^] | 11.8 (0.2) | 12.7 (1.4) | 12.7 (3.5) | 12.8 (2.0) | 12.9 (3.2) | 11.5 (1.6) | 12.2 (0.1) | 12.3 (1.1) | 11.5 (1.9) | 11.5 (3.3) | 12.3 (2.6) | 12.7 (2.5) | 12.5 (3.4) | 12.2 (2.2) | 11.8 (1.4) | 11.2 (1.2) | 12.6 (2.8) | 12.4 (2.1) | 11.8 (2.1) | 11.6 (2.3) |  |
| Cr [mg kg^-1^] | 67.6 (2.2) | 73.3 (4.4) | 70.8 (5.2) | 72.5 (2.8) | 72.7 (7.1) | 67.4 (7.5) | 70.6 (0.2) | 69.2 (1.6) | 65.9 (1.7) | 64.0 (5.9) | 70.3 (3.1) | 71.9 (2.1) | 73.2 (7.0) | 68.9 (4.0) | 63.7 (0.6) | 66.3 (3.0) | 72.1 (1.7) | 73.1 (2.0) | 66.3 (2.4) | 65.0 (1.4) |  |
| Cu [mg kg^-1^] | 40.8 (3.0) | 40.0 (2.1) | 52.0 (4.0) | 44.4 (1.3) | 44.1 (2.6) | 33.1 (1.8) | 33.2 (1.0) | 33.2 (2.1) | 28.9 (2.4) | 26.8 (4.1) | 37.4 (2.0) | 37.5 (0.6) | 36.6 (1.4) | 36.0 (0.3) | 29.4 (3.6) | 36.3 (1.1) | 35.7 (0.7) | 34.9 (1.3) | 30.5 (0.4) | 28.3 (2.9) |  |
| Ni [mg kg^-1^] | 49.1 (1.0) | 52.4 (1.4) | 51.3 (2.4) | 52.0 (1.6) | 52.1 (1.8) | 47.5 (1.4) | 49.6 (0.5) | 50.5 (0.9) | 47.4 (1.4) | 46.4 (3.2) | 50.2 (1.7) | 51.8 (0.4) | 51.7 (2.3) | 50.5 (1.3) | 47.2 (1.2) | 47.0 (1.2) | 51.1 (0.6) | 51.2 (1.3) | 47.9 (1.4) | 47.3 (2.1) |  |
| Pb [mg kg^-1^] | 21.7 (0.7) | 23.0 (5.4) | 23.1 (5.7) | 24.6 (24.6) | 23.0 (9.2) | 20.7 (3.6) | 21.0 (4.1) | 21.7 (2.2) | 19.5 (3.7) | 17.5 (1.6) | 32.2 (30.0) | 26.5 (20.8) | 30.7 (37.2) | 25.7 (18.8) | 20.1 (0.8) | 24.4 (3.9) | 24.7 (12.8) | 24.1 (4.6) | 21.7 (15.7) | 19.4 (3.5) |  |
| Sb [mg kg^-1^] | 1.3 (26.5) | 1.4 (8.8) | 1.4 (15.6) | 1.4 (28.4) | 1.4 (5.8) | 1.3 (9.3) | 1.2 (7.5) | 1.2 (10.0) | 1.3 (3.7) | 1.3 (25.8) | 1.4 (6.7) | 1.5 (12.6) | 1.3 (6.1) | 1.3 (9.1) | 1.1 (21.5) | 1.3 (12.5) | 1.4 (6.7) | 1.5 (8.4) | 1.3 (13.8) | 1.3 (9.5) |  |
| Sn [mg kg^-1^] | 2.2 (5.9) | 2.2 (9.4) | 2.0 (13.2) | 2.1 (4.7) | 2.1 (14.2) | 2.1 (4.0) | 1.9 (5.2) | 1.9 (2.7) | 1.8 (7.5) | 1.8 (7.8) | 2.2 (7.8) | 2.1 (8.9) | 2.0 (7.7) | 1.9 (6.6) | 1.7 (9.3) | 2.0 (2.8) | 2.0 (2.6) | 2.0 (7.2) | 1.9 (1.7) | 1.9 (6.6) |  |
| V [mg kg^-1^] | 58.9 (2.9) | 63.2 (6.0) | 61.9 (7.1) | 64.1 (3.0) | 64.3 (10.1) | 56.1 (4.8) | 60.3 (0.4) | 59.0 (2.5) | 55.9 (2.7) | 53.5 (7.1) | 60.9 (3.2) | 62.0 (3.6) | 63.6 (9.0) | 58.6 (5.0) | 52.5 (2.0) | 56.6 (1.8) | 62.4 (0.9) | 62.6 (4.7) | 58.1 (3.5) | 56.3 (1.8) |  |
| Zn [mg kg^-1^] | 79.9 (1.8) | 80.2 (3.4) | 76.5 (4.8) | 78.0 (1.1) | 76.8 (2.3) | 74.3 (2.1) | 73.0 (1.3) | 72.7 (2.6) | 67.0 (0.4) | 63.9 (3.9) | 78.3 (4.0) | 76.0 (0.5) | 74.4 (2.4) | 71.9 (1.2) | 67.4 (1.4) | 80.5 (0.5) | 79.3 (0.8) | 84.4 (1.6) | 78.0 (1.4) | 72.0 (0.7) |  |
| B [g kg^-1^] | 0.05 (4.8) | 0.05 (8.3) | 0.04 (9.7) | 0.05 (4.0) | 0.05 (12.0) | 0.043 (7.6) | 0.05 (0.3) | 0.04 (3.1) | 0.04 (4.7) | 0.04 (8.6) | 0.05 (4.7) | 0.05 (4.8) | 0.05 (11.3) | 0.04 (7.9) | 0.04 (3.6) | 0.04 (1.6) | 0.05 (2.4) | 0.04 (6.4) | 0.04 (6.1) | 0.04 (2.3) |  |
| Mo [mg kg^-1^] | 0.3 (31.6) | 0.3 (15.9) | 0.3 (17.2) | 0.3 (0.09) | 0.3 (17.4) | 0.2 (21.6) | 0.3 (17.3) | 0.3 (43.1) | 0.2 (0.24) | 0.3 (17.5) | 43.3(0.8) | 45.0 (1.4) | 42.7 (1.5) | 42.0 (1.1) | 48.4 (1.9) | 38.2 (0.7) | 42.3(1.7) | 41.6 (1.6) | 49.9(1.2) | 54.0 (0.7) |  |
| Ca [g kg^-1^] | 44.9 (0.7) | 42.9 (1.4) | 42.8 (3.1) | 43.4 (1.2) | 43.8 (0.7) | 42.6 (0.7) | 42.5 (0.1) | 42.8 (2.4) | 49.9 (1.4) | 56.0 (2.2) | 26.0 (1.5) | 26.7 (0.7) | 26.6 (2.3) | 26.0 (1.0) | 23.7 (1.6) | 24.1 (1.5) | 26.5 (0.7) | 26.6 (1.3) | 25.9 (1.2) | 25.1 (0.9) |  |
| Fe [g kg^-1^] | 25.4 (1.0) | 26.9 (1.3) | 26.6 (2.1) | 26.8 (1.1) | 26.9 (1.3) | 23.9 (1.9) | 25.7 (0.7) | 26.1 (1.2) | 24.8 (0.7) | 24.4 (3.3) | 7.6 (4.7) | 7.7 (6.4) | 7.9 (15.9) | 6.9 (8.6) | 6.5 (5.2) | 7.1 (2.2) | 7.6 (2.5) | 7.5 (8.3) | 7.1 (5.8) | 7.0 (2.4) |  |
| K [g kg^-1^] | 7.4 (5.6) | 7.7 (10.2) | 7.5 (11.3) | 7.9 (4.2) | 7.9 (17.4) | 7.1 (8.3) | 7.6 (0.7) | 7.0 (2.3) | 6.9 (5.3) | 6.7 (9.5) | 9.7 (2.5) | 9.8 (1.5) | 9.6 (3.7) | 9.2 (1.7) | 8.7 (1.5) | 9.0 (1.1) | 9.6 (0.5) | 9.4 (2.3) | 9.4 (1.2) | 9.5 (0.7) |  |
| Mg [g kg^-1^] | 9.5 (1.1) | 9.8 (2.8) | 9.7 (3.2) | 9.8 (1.5) | 9.7 (3.7) | 9.2 (1.7) | 9.5 (0.5) | 9.4 (1.8) | 9.3 (1.0) | 9.4 (4.5) | 0.55 (2.1) | 0.65 (5.4) | 0.63 (1.6) | 0.64 (4.2) | 0.63 (0.4) | 0.46 (2.6) | 0.69 (5.5) | 0.65 (5.7) | 0.57 (2.4) | 0.56 (3.4) |  |
| Mn [g kg^-1^] | 0.52 (2.5) | 0.68 (0.9) | 0.73 (4.6) | 0.66 (1.8) | 0.64 (4.9) | 0.44 (2.2) | 0.54 (2.5) | 0.63 (1.1) | 0.58 (3.0) | 0.57 (2.3) | 0.2 (0.1) | 0.2 (0.1) | 0.2 (0.1) | 0.2 (0.2) | 0.3 (17.3) | 0.3 (17.3) | 0.3 (15.6) | 0.3 (15.6) | 0.3 (0.2) | 0.3 (0.2) |  |
| P [g kg^-1^] | 0.71 (1.1) | 0.61 (2.1) | 0.55 (3.1) | 0.62 (0.7) | 0.63 (2.1) | 0.60 (2.3) | 0.54 (0.9) | 0.49 (2.9) | 0.40 (1.9) | 0.36 (3.9) | 0.61 (2.2) | 0.57 (0.4) | 0.80 (49.7) | 0.55 (0.6) | 0.46(2.0) | 0.73 (1.1) | 0.65 (3.4) | 0.69 (2.5) | 0.45 (3.7) | 0.40 (5.2) |  |
| S [g kg^-1^] | 0.51 (1.6) | 0.24 (7.6) | 0.23 (16.7) | 0.25 (11.5) | 0.24 (5.9) | 0.60 (3.2) | 0.36 (6.9) | 0.25 (14.5) | 0.17 (1.5) | 0.17 (4.5) | 0.42 (3.3) | 0.28 (4.3) | 0.21 (2.9) | 0.20 (0.8) | 0.20 (2.7) | 0.63 (3.6) | 0.21 (5.9) | 0.19 (1.9) | 0.15 (2.0) | 0.15 (1.1) |  |
| Al [g kg^-1^] | 34.4 (4.0) | 36.7 (8.2) | 36.4 (9.4) | 37.9 (3.5) | 38.0 (13.5) | 33.1 (6.4) | 35.9 (0.8) | 34.7 (2.0) | 33.9 (3.8) | 32.7 (8.0) | 35.7 (4.3) | 36.3 (5.1) | 37.7 (11.8) | 34.1 (7.0) | 31.0 (3.1) | 32.8 (1.9) | 36.5 (1.1) | 36.8 (6.2) | 35.0 (4.2) | 34.4 (1.5) |  |
| Na [g kg^-1^] | 0.62 (11.8) | 0.61 (20.6) | 0.61 (17.0) | 0.78 (9.5) | 0.78 (28.5) | 0.66 (12.0) | 0.69 (0.9) | 0.61 (2.9) | 0.67 (1.9) | 0.72 (3.9) | 0.70 (2.2) | 0.68 (0.4) | 0.74 (49.7) | 0.62 (0.6) | 0.64 (2.5) | 0.56 (1.1) | 0.61 (3.4) | 0.64 (2.5) | 0.65 (3.7) | 0.73 (5.2) |  |

**Figure SM.1** Average daily temperature during the experimental period. Eight-week average temperature: 8.3 °C.

**Figure SM.2** Concentration of imidacloprid, dimethomorph and glyphosate + AMPA in the SFCW water (mg L^-1^) and the upper 5-cm of topsoil (mg kg^-1^ soil dw) samples for each sampling position of the SFCW. LOQ (limit of quantification = 0.05 mg L^-1^ water or mg kg^-1^ soil dw) of each single pesticide and AMPA is reported.

**LOQ**

**LOQ**

**SM.3 Infrared spectra of soil-glyphosate complex**

A solution of 2 g L^-1^ (11.8 mM) glyphosate was prepared by dissolving 10 mg of glyphosate (98% PESTANAL, Sigma Aldrich) in 5 mL milliQ® water (Millipore) in a 10 mL Eppendorf tube. The solution was stirred for 1 min and then sonicated for 5 min until clear. Then, the glyphosate solution pH was brought to 8 by adding a few drops of freshly prepared 0.1 NaOH aqueous solution, to replicate the pH value of CW water.

A sample of 100 mg of wet SFCW soil was added with 1 mL of 2 g L^-1^ glyphosate solution at pH 8 into a centrifuge tube and gently shaken overnight onto a horizontal shaker. The soil suspension was then centrifuged at 2800 *g* for 25 min. The supernatant was collected and analysed for glyphosate content (resulting 42% of added amount), and the soil pellet was dried at RT in a desiccator under reduced pressure for 24 h. In these conditions, the mass ratio of glyphosate retained by soil dry weight was 0.6:10. Control soil was exposed to the same sequence of treatments, except for the contact with a water solution at pH 8 instead of the glyphosate solution at the same pH value. IR analysis were performed on pellets of control soil and glyphosate-treated dried soil samples.

Samples of dried soil (2.5 mg each) were thoroughly mixed with 15 mg of KBr (Sigma-Aldrich, IR spectroscopy grade). Pellet of each sample was obtained by mechanical press (Specac) at ca. 7 tons cm^2^ and placed into an IR cell equipped with KBr windows permanently attached to a vacuum line (residual pressure 5x10^-2^ mbar). Finally, FT-IR spectra of all the pellets were collected on a TENSOR 27 (Bruker) with 4 cm^-1^ resolution and 64 scans.

*Description of the CW water spectrum reported in Figure 6.*

In short, the broad absorbance between 3800 and 2700 cm^-1^ is mainly ascribable to stretching modes of -OH of hydration water molecules of ionic species, as well as -OH of phenols, alcohols, silanols and -NH^+^ of amines in organic molecules. A relevant contribution of carboxylic -OH groups to the signal was ruled out by the alkaline pH of the medium. The stretching modes of -CH groups of aliphatic and aromatic structures are not visible as masked by the tail at low wavenumbers of the broad band. In the 1770-1600 cm^-1^ region, bending of -OH (mainly hydration water molecules) and protonated amine groups, together with stretching of carbonyl groups of ketons, amides, quinones, and esters, are found. The 1600-1450 cm^-1^ region is typical of asymmetric stretching of carboxylates whereas that at 1450-1250 cm^-1^ of stretching of carboxylates (symmetric) and carbonates, as well as bending of phenols -OH. In the 1250-950 cm^-1^ region bending of keton groups and stretching of C-O of phenols groups are visible. Finally, the band at 862 is cm^-1^ is typical of bending modes of carbonates but also of nitrates and sulphates.

**FigureSM.3** FT-IR spectrum of glyphosate adsorbed on the SFCW soil (Gly-soil). Spectrum of control soil is reported for comparison.
